# Toledo Medical Association

**Published:** 1877-10

**Authors:** 


### TOLEDO MEDICAL ASSOCIATION.

---

Meeting of August 17th, 1877.

Cyrus A. Kirkley, M.D., in the chair. C. S. Chamberlin, Secretary.

Dr. Hathaway, the regular essayist, presented a paper "*On Some of the Therapeutic Phases of Bowel Complaints.*"

Dr. Ridenour remarked on the importance of indigestion as an element in cholera infantum, even in the adult a short attack of acute enteritis will arrest this function for several days.

Dr. Reed asked what his treatment would be in cases of young children with constant watery green cheesy discharges.

Dr. R. replied, small doses of mercury.

Dr. Hathaway, in reply to the discussion, remarked that he had not given ipecac. in homeopathic doses, but sufficient doses to cause emesis, at first, and afterward in small doses, when tolerance had become established. When there is much mucus in the discharges, gives aloes; if acid, he combines alkalies. Is impressed with the necessity of observing the condition of the superficial capilaries; belladonna is beneficial by stimulating this circulation, and thus curing the bowel trouble. Digestion is always affected by bowel troubles, even in dysentery.

Dr. Jones reported the case of a lady aged 32, affected with tuberculosis, who early last spring had hectic, and died last week. The case was remarkable for the emaciation, which was out of all proportion to the local lesion. Even for three months before death her features were pinched. The post-mortem revealed the mesentery studded with large glands, the size of a pigeon's egg, and less, (the specimens exhibited). There was no pain, tympanitis or inflammation of the abdomen, pointing to their existence. The lungs were also involved.

Dr. Thorn remarked on the firm union existing between the pleura. She had had great indigestion for years; so much so that lesion of the stomach was suspected. Some dysentery also existed; eroding ulcers were found in the colon.

Dr. Chamberlin referred to a case of a young lady, who although eating heartily, steadily emaciated and died. The case reported throws much light on such cases.

Dr. Thorn reported case of abdominal dropsy in a young man at the St. Vincent's Hospital. Examination found impeded heart action. He tapped him and drew 36 pints, and two days after 6 pints more. He is now filling again.

Dr. Cherry saw the patient early in his sickness. Jaundice existed at that time. He strongly suspected liver disease, probably malignant.

Dr. Reed objected to early tapping in ascites, and reported a case where the condition seemed hopeless, which got well on quinia and iron, with large doses of comp. jalap powder.

Dr. Ridenour reported a case where gas was mistaken for fluid, and dry tapping was performed.

Dr. Jones believed tapping cures more frequently than other means.

Dr. Curry remarked on elaterium often curing after every other remedy fails.

Dr. Thorn replied that he regarded the liver of this patient as being diseased.

Dr. Reed reported case of scalp tumor on new born child delivered by a midwife. Showed evidence of suppuration. It was evacuated, broken down blood being freely discharged. Subsequently, the sublingual glands enlarged and discharged. Child died on the twelfth day.

Dr. Forbes remarked that if the scalp tumor had not been opened, there would have been no subsequent septicæmia.

Dr. Waddel reported case of lady aged 38, who was taken with a slight pain in the abdomen, gradually increasing until it became, after several days, very severe. Near the umbilicus an indurated space could be felt. Iodine tinc. was freely applied, and "Glyconated Emulsion of Cod Liver Oil" given internally. The patient soon recovered. Diagnosis was somewhat obscure, but it was regarded as mesenteric trouble.

Dr. Collimore reported case of poisoning by bitter apple, taken for the purpose of causing abortion. The infusion of a whole apple was taken at night. It first caused drastic, soon running into bloody discharges from the bowels, then vomiting and dyspnœa. In 24 hours after taking, at which time he first saw the young woman, the breathing was very difficult, no tenderness of the abdomen, and little change in the pulse. Opium was freely given, but the vomiting did not cease for 24 hours after. He had observed three other cases of poisoning from this drug, but none so severe.

Dr. Chamberlin reported case of remarkable umbilical hemorrhage preceded by a pustular eruption on the palms of the hands and soles of the feet, a few days after birth. It nursed well for several days. The bleeding was finally arrested by passing a surgical needle through the cord and applying figure 8. Blood then began to flow from pustules until death. In thirty years' practice he has seen three cases of excessive umbilical hemorrhage, one of which died.

Meeting adjourned.

